# Developing, Piloting and Evaluating a Patient Support Portal for Men With Prostate Cancer in Victoria: An Action Research Study

**DOI:** 10.1111/hex.70149

**Published:** 2025-01-19

**Authors:** Benjamin Shemesh, Jacinta L. Opie, Rodney L. Dunn, Garth Mclaughlin, Vivek Argawal, Amanda Pomery, Chris Mac Manus, Colin O'Brien, Katrina Lewis, Max Shub, Paula Wilton, Prassannah Satasivam, Melanie Evans, Jeremy Millar, Sue M. Evans

**Affiliations:** ^1^ School of Public Health and Preventive Medicine Monash University Melbourne Victoria Australia; ^2^ Department of Urology University of Michigan Ann Arbor Michigan USA; ^3^ Movember Melbourne Victoria Australia; ^4^ Consumer Representatives Melbourne Victoria Australia; ^5^ Medical Services Division Alfred Health Melbourne Victoria Australia; ^6^ eHealth Division Victorian Department of Health Melbourne Victoria Australia; ^7^ Department of Urology Northern Health Melbourne Victoria Australia; ^8^ Department of Surgery, Melbourne Medical School, Northern Health The University of Melbourne Melbourne Victoria Australia; ^9^ Victorian Cancer Registry Cancer Council Victoria Melbourne Victoria Australia

**Keywords:** comparator, patient portal, patient‐reported outcomes, prostate cancer, supportive care, tool

## Abstract

**Introduction:**

Men with prostate cancer (PCa) and their support providers face challenges in accessing high‐quality, impartial information tailored to their specific needs to enhance their overall care and decision‐making. We describe the development, piloting and evaluation of the co‐designed web portal ‘BroSupPORT’.

**Methods:**

IT teams developed and integrated BroSupPORT into the Victorian Prostate Cancer Outcomes Registry (PCOR‐Vic) electronic patient‐reported outcome follow‐up process. A comparator tool was built enabling men to compare their patient‐reported outcome results against men of similar age, risk profile and after the same treatment. PCOR‐Vic participants were invited to access BroSupPORT after 12 months of follow‐up patient‐reported outcome measure completion. Factors associated with consent to BroSupPORT were determined using logistic regression. Portal access data were gathered from PCOR‐Vic data extracts and Google Analytics. A survey on portal exit and 2 weeks after consent was used to collect feedback.

**Results:**

Over a 4‐month pilot, 331/583 (57%) men consented to accessing BroSupPORT. Among those men who accessed the portal, the majority (209/331 =63%) were diagnosed in a private hospital and resided in a major city (214/331=65%). On average, men spent 3:20 min on the portal, with sexual function aspects receiving the most attention. Twenty‐three percent of men revisited the portal during the pilot. Most men found the portal easy to use, reassuring and informative, while 9% found the patient‐reported comparisons difficult to interpret.

**Conclusion:**

A patient portal—enabling men to compare their patient‐reported outcomes with other similar men and providing access to information and resources—may be a scalable solution in addressing the complex supportive care needs of men with PCa on a population basis.

## 1 Introduction

A population‐based registry collecting patient‐reported outcomes (PROs) from men with prostate cancer can provide a rich epidemiological impression of survivorship problems as well as assist individual men. It is widely known that prostate cancer (PCa) treatment can result in bothersome urinary, sexual and bowel symptoms that can have a significant impact on quality of life (QoL) [1, 2, 3, 4, 5, 6]. Provider assessments may differ sharply from patient assessments, so PROs offer a unique opportunity to enhance patient‐centred care by capturing health‐related QoL (HRQoL) from the perspective of the patient [7, 8, 9, 10].

The Victorian Prostate Cancer Outcomes Registry (PCOR‐Vic), established in 2009, collects both clinical information and PROs [11]; PROs are collected 12 months after treatment using the Expanded Prostate Cancer Index Composite (EPIC‐26) [12], a widely used, standardised, valid, reliable, patient‐completed instrument that evaluates patient function and bother after PCa treatment. This information has been fed back as aggregate benchmark reports to hospitals and at the patient level to clinicians to prompt action to address identified QoL issues and improve service delivery. PCOR‐Vic did not previously provide men with individual feedback. Patient ‘web‐portals’ might enable an innovative opportunity to engage men in self‐managing their disease by integrating PROs to prompt health‐seeking behaviour.

Research recently undertaken by this research team used co‐design methodology to develop content for an information portal and to ascertain the views of men and support providers on the concept of a comparator tool, which enables men to compare their quantified PROs with comparable men [13]. The idea for a comparator tool developed from discussions with men during the research team's collection of PROs. Many men inquired whether their symptoms were ‘normal’ and sought information specific to the issues highlighted in their survey responses. Although patient portals for PCa that incorporate PRO symptom tracking as well as the ability to compare PROs have been developed in the United States [14], no evaluation has been published within an Australian population that assesses acceptability, user experience (UX) and impact on addressing supportive care needs, and there is little evidence about their utility in supporting men with PCa [14, 15].

The aim of this study was to describe the characteristics of users of a patient portal designed to help men manage their disease and treatment side effects, and to assess the acceptability of a tool that compares their QoL with that of other men with PCa. The objectives were to (1) describe the development of a comparator tool and patient portal; (2) assess the characteristics of men accessing the portal; (3) detail portal activity during the pilot; and (4) describe men's perceptions of the PRO comparator tool and the portal. As this was a novel intervention and acceptability of the PRO comparator had not been previously evaluated at a population level, we did not embark on this study with an expectation of what success would look like.

## 2 Materials and Methods

### 2.1 Study Design

**Figure 1:**
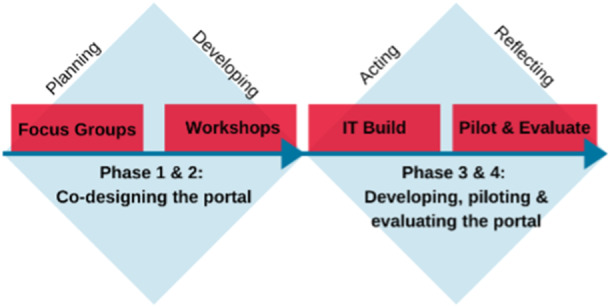
The action research process informing the development of BroSupPORT.

This study adopted an ‘action research’ process to develop the online portal. Action research consists of four main phases—planning (identifying the issue/topic); developing (developing the intervention/action); action (implementing the intervention/action); and reflecting (evaluating, communicating and refining) [16]. Phases 1 and 2 of the study were completed in October 2020, with findings previously published [13]. This study focuses on phases 3 and 4 of the action research process, which incorporates work related to information technology (IT) development, pilot and evaluation (Figure 1). Based on men's preferences regarding the content and format of the information displayed within an online portal, we iteratively developed a PCa support portal named ‘BroSupPORT.’ The content and layout of the BroSupPORT portal, which were previously conceptualised through co‐design workshops, were developed as representative prototypes.

### 2.2 Setting

**Figure 2:**
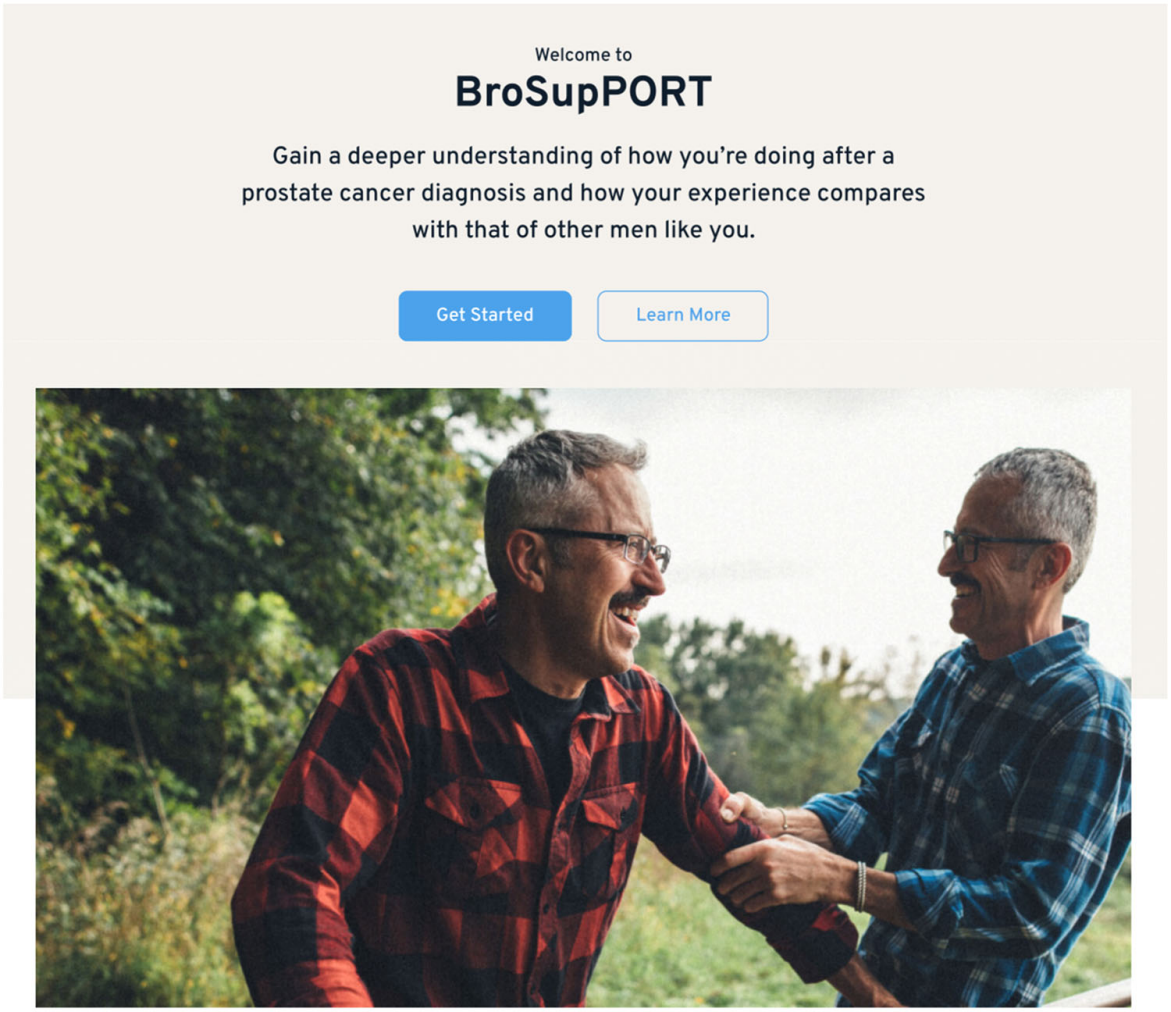
The BroSupPORT landing page.

The BroSupPORT portal was piloted in a population of Victorian men from April to July 2021 (Figure 2 shows the BroSupPORT Landing Page). The PCOR‐Vic portal was modified to enable men to consent to participate in the BroSupPORT project after they had completed their routinely collected PRO survey 12 months after diagnosis/initial treatment [11]. After men consented to the study, their demographic, diagnosis and treatment details and EPIC‐26^12^ responses were extracted in a de‐identified format from the PCOR‐Vic database. These files were transferred via a secure file transfer protocol over transport layer security to Movember servers, which in exchange sent back a unique token that was then provided to men as an email link to access the portal. Each unique token provided access for 30 days, after which men would no longer be able to access the portal to ensure the safety of their data. Men were encouraged to save the comparison information to utilise the data beyond 30 days.

Men with PCa who participated in these co‐design workshops during phase 1 of the project (*n* = 6) and members of the consumer advisory group (*n* = 3) were engaged to participate in a series of UX design interviews. Interviews (*n* = 9 total) were conducted by a team consisting of a UX design expert, a web developer and the BroSupPORT Project Officer and enabled the team to identify improvements to prototype usability. Men with PCa who participated in co‐design workshops were also invited to participate in user acceptance testing (UAT) to verify the portal before going live. Men (*n* = 6) were sent a UAT checklist, developed by the project team, containing various tasks to perform on the portal to determine whether the portal was ready to be deployed.

Google Analytics was used to understand how long men spent on the portal, how many and which articles were viewed and whether or not they saved any pages. An initial feedback survey was administered to men who accessed the BroSupPORT portal using HotJar [17] when exiting the Movember TrueNorth website. This asked men whether they found the comparator tool useful, whether it was easy to understand the information provided, whether the articles they read were useful and whether men were likely to take action based on the information within the portal. A second survey, administered using Qualtrics, asked men their age and most recent treatment for PCa, whether they accessed the BroSupPORT website, whether they viewed the comparison information, whether they viewed the supporting articles, whether they watched the video stories and whether they found the video stories helpful. This was sent 2 weeks after study consent to the email address nominated by men at the time of completion of the PROM.

### 2.3 Participants

Men were eligible for recruitment to the pilot study if they spoke English, responded to telephone contact from PCOR‐Vic research staff and subsequently completed their PROM electronically via email. Men were not followed up if they declined to consent when prompted to do so after completing the PROM. Recruitment was monitored weekly to determine the percentage of men completing the PROM who consented to participate in BroSupPORT.

### 2.4 Variables

A data model was developed based upon preexisting data of 11,000 men with PCa contributing to the PCOR‐Vic for the years 2016–2021 to allow men to see how their EPIC‐26^12^ survey responses compared to similar men with PCa in Victoria. EPIC‐26^12^ measures HRQoL urinary, bowel, sexual and hormonal domains. EPIC‐26^12^ statistical modelling was undertaken to determine expected values, adjusted for patient and treatment characteristics for each of the study outcomes. Treatment and age were mandatory fields in the model because they were considered clinically necessary to provide useful estimates. Treatment values used in the model included surgery, radiation therapy (including external beam and brachytherapy) androgen deprivation therapy with and without chemotherapy, active surveillance (AS), watchful waiting (WW) and other treatments. Men who had surgery and then subsequent treatments were categorised as surgery. Likewise, men who had radiation therapy and subsequent treatments were categorised as radiation therapy. The model was constructed with and without inclusion of the National Comprehensive Cancer Network (NCCN) risk level [18]. Other factors considered but ultimately not included in the model were clinical N‐stage and M‐stage. Linear regression models were used to produce the adjusted estimates for the EPIC domain scores. The EPIC‐26^12^ domains were dichotomised into either no problem or any problem (very small, small, moderate or large). Logistic regression models were used for each of the dichotomous outcomes to produce estimates of the better of the two dichotomised values. Two sets of final models were created, one containing only the required factors (treatment and age) and the other including those plus the NCCN risk level. Using each of these final models separately, model estimates were produced for each outcome based on every permutation of treatment, every integer age between 30 and 100, and each of the five NCCN risk levels. The PRO comparator tool would use the model based on treatment, age and NCCN if the NCCN risk level was known for a patient; otherwise, the tool would use the value based on the model containing only treatment and age, provided they were known.

Data extracted from the PCOR‐Vic database included participants’ demographic details (age, postcode, suburb), treatment details, NCCN risk group, diagnosing institution and diagnosis date. Postcode of residence was used to geocode coordinates, which were mapped to Statistical Area Level 2 (mean population size of 10,000 persons, defined by the Australian Bureau of Statistics [ABS]). The ABS Index of Relative Socioeconomic Disadvantage was used to determine area‐level socioeconomic disadvantage (Socio‐Economic Indexes for Areas [SEIFA]), grouped into quintiles [19]. Continuous variables are summarised as medians and interquartile ranges, and categorical variables are summarised as frequencies and proportions.

### 2.5 Statistical Methods

**Figure 3:**
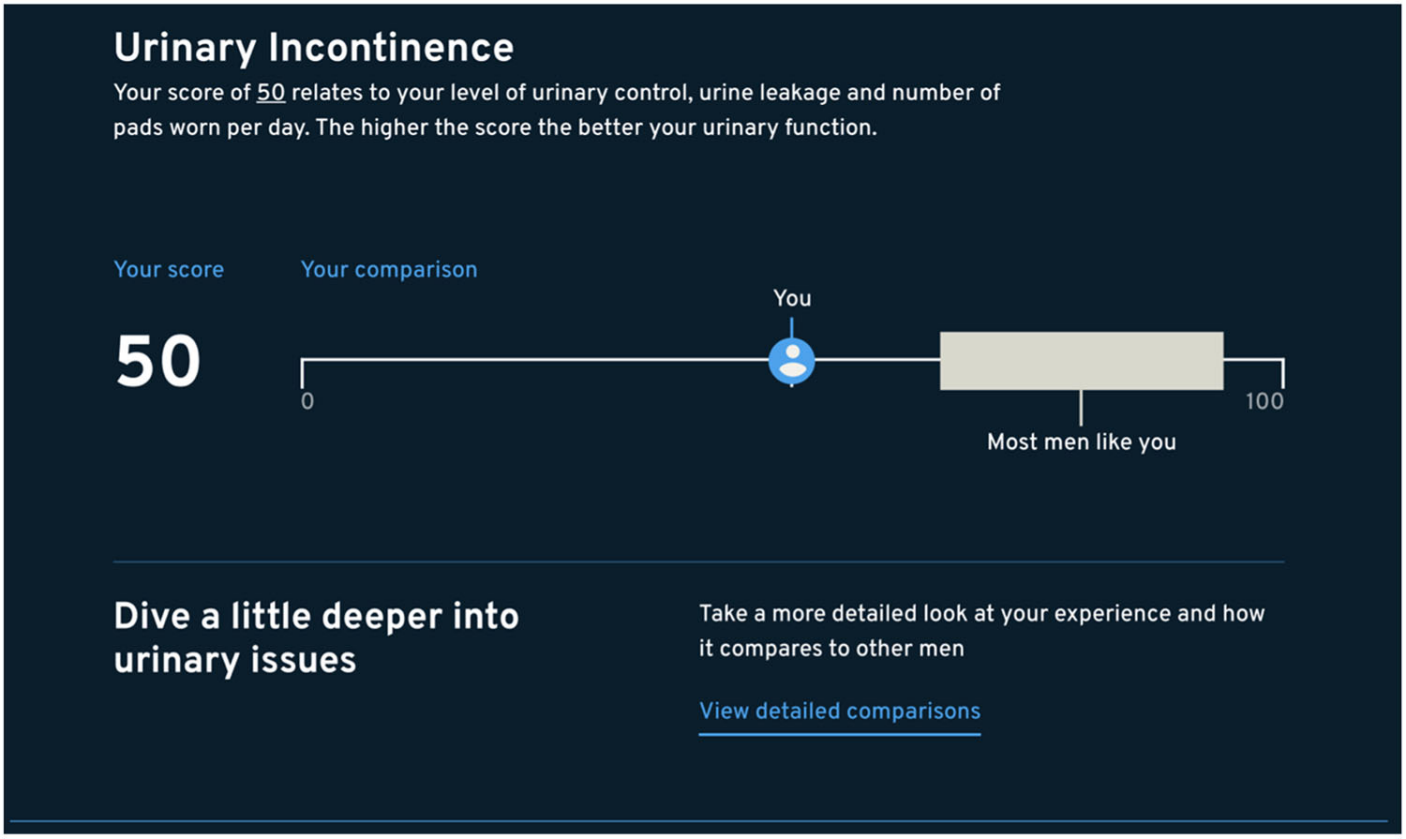
Example of the PRO comparator tool for urinary incontinence.

**Figure 4:**
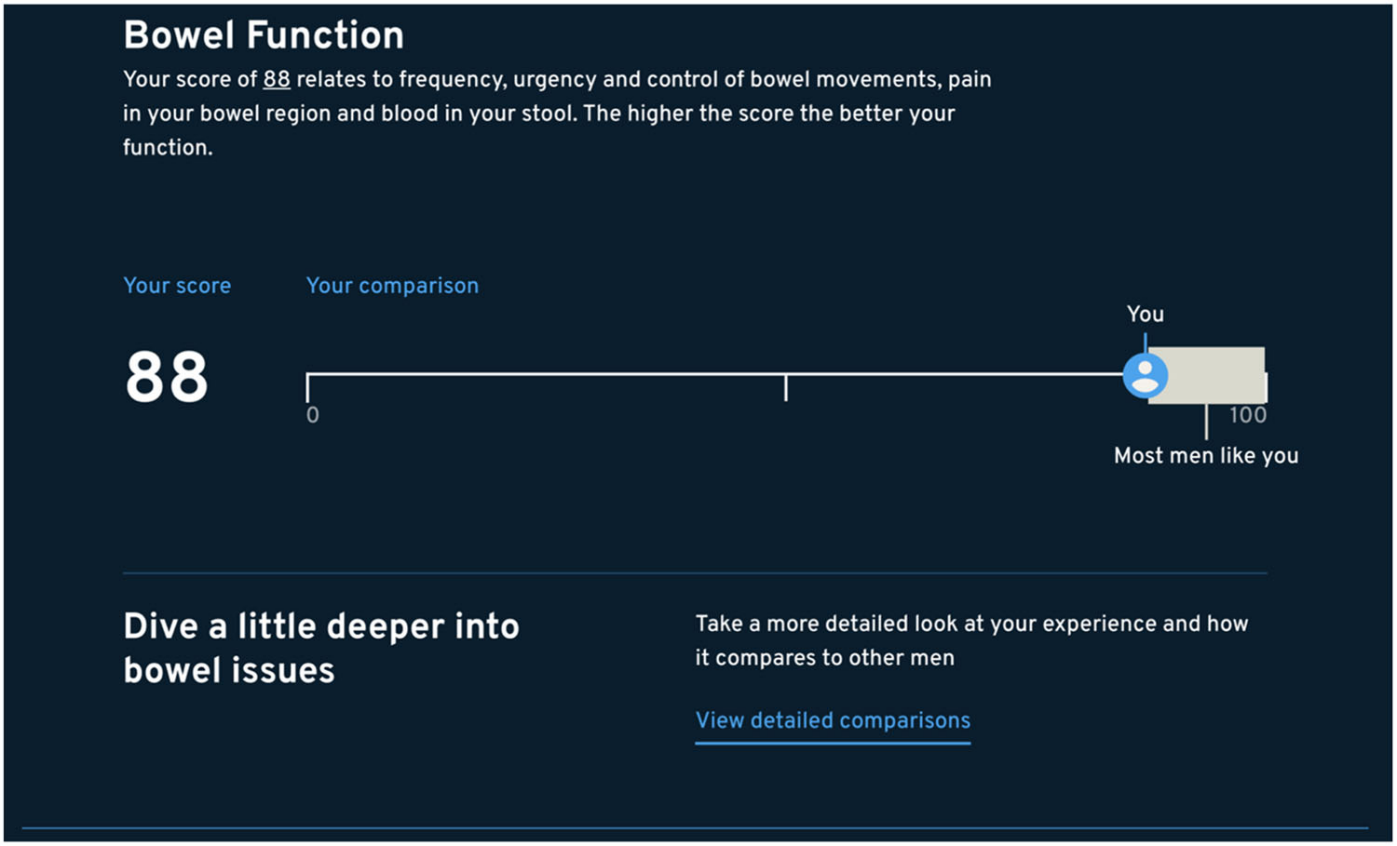
Example of the PRO comparator tool for Bowel Function.

The EPIC‐26^12^ responses were scored using the scoring instructions for the EPIC‐26 survey^12^. Responses are transformed into five domain scores scaled from 0 to 100, with a higher score indicating better function (Figures 3 and 4). Domain scores were set to missing if the number of non‐missing items fell below 80% of the items in each domain as per EPIC‐26^12^ scoring instructions. EPIC‐26^12^ scores were compared between consented users and non‐participants. Multivariable logistic regression models were used to determine variables associated with consent status utilising backward selection to arrive at a parsimonious model. This approach was used as it is relatively efficient in removing variables that are not correlated with the outcome of consent status. Survey feedback analyses were undertaken using descriptive statistics and thematic analysis, with similar responses clustered together to identify common themes. Statistical analyses were performed using SAS version 9.4 (SAS Institute, Cary, North Carolina, United States). This study was approved by the Monash University Human Research Ethics Committee (no. 24688).

## 3 Results

### 3.1 Participants

**Figure 5:**
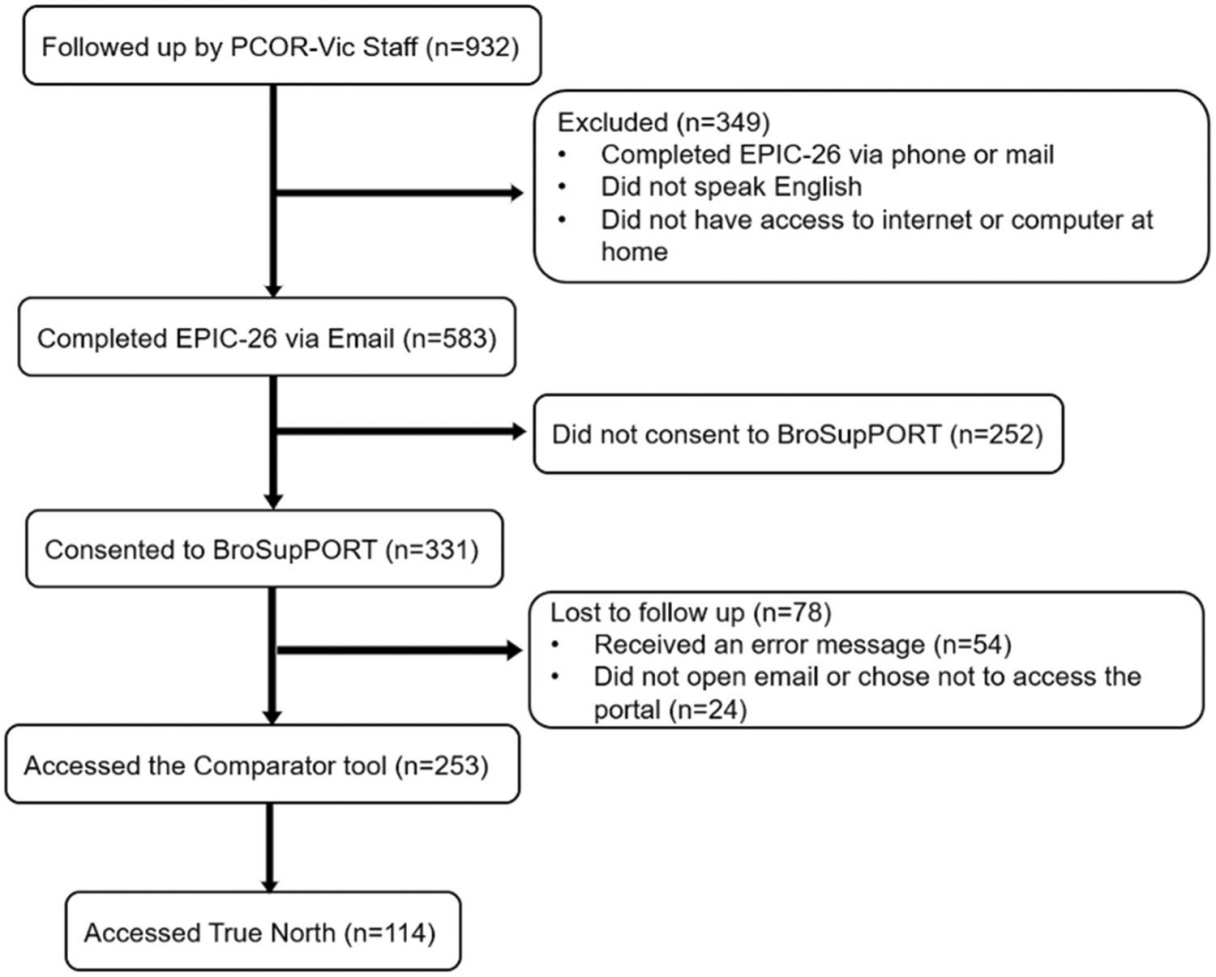
CONSORT diagram of the BroSupPORT Pilot (April‐July 2021).

This study was conducted between December 2020 and August 2021, and the pilot period for the portal was from April 2021 to July 2021. During this period, PCOR‐Vic attempted to follow up with 932 men 12 months after treatment or diagnosis for men on AS or WW. Of these, 583 (63%) men completed the EPIC‐26^12^ questionnaire. Fifty‐seven percent (*n* = 331) consented to accessing BroSupPORT (Figure 5). Characteristics of men consenting to participate in the BroSupPORT study were compared with the distribution of men according to these characteristics as reported by the population‐based Victorian Cancer Registry (VCR) in 2019. The median age of men was 68 years. Of the men who accessed BroSupPORT (209/331 = 63%), most were diagnosed in a private hospital (209/331, 63%) and resided in a major city (214/331 = 65%) (Table 1). The almost 2:1 ratio of men who were diagnosed in private hospitals consenting to participate reflects the distribution of men recruited to PCOR‐Vic from private hospitals. The distribution of men residing in outer regional and remote areas of Australia who participated in the study was similar to the distribution of men diagnosed with PCa in Victoria in 2019 (7% vs. 5%). However, men participating in the study were less disadvantaged (Index of Relative Socioeconomic Advantage and Disadvantage) when compared to the VCR (*p *= 0.005). Half of the men (*n* = 164) consenting to access BroSupPORT had surgery as their primary treatment, and one‐third (*n* = 108) were classified as having intermediate‐risk disease.

**Table 1:** Characteristics of men consenting/not consenting to participate in BroSupPORT and comparison with characteristics of the Victorian population diagnosed with prostate cancer reported to the Victorian Cancer Registry.

	Consented users	Non‐consented users	*p*	Victorian Cancer Registry participants (2019)
	*N* (%)	*N* (%)		*N* (%)
	(*n* = 331)	(*n* = 252)		(*n* = 6034)
Age group			0.5	
≤ 59	50 (15%)	34 (13%)		927 (15%) [< 60 age group]
60–64	47 (14%)	50 (20%)		900 (15%)
65–69	86 (26%)	66 (26%)		1376 (23%)
70–74	78 (24%)	57 (22%)		1234 (20%)
≥ 75	70 (21%)	47 (19%)		1597 (26%)
Diagnosing institute			0.04	
Public	114 (34%)	78 (31%)		N/A
Private	209 (63%)	164 (66%)		
Unknown	3 (1%)	4 (2%)		
Location of residence			0.08	
Major cities	214 (65%)	163 (65%)		4212 (70%)
Inner regional	94 (28%)	66 (26%)		1480 (25%)
Outer regional and remote	23 (7%)	23 (9%)		321 (5%)
Missing	0	0		21
Socio‐economic iIndexes for areas index for relative socio‐economic disadvantage			0.005	
1 Most disadvantaged	19 (6%)	25 (10%)		1027 (17%)
2	55 (17%)	37 (15%)		1072 (18%)
3	60 (18%)	52 (21%)		1199 (20%)
4	70 (21%)	39 (15%)		1200 (20%)
5 least disadvantaged	127 (38%)	99 (39%)		1492 (25%)
Missing	0	0		44
Treatment type			0.9	N/A
Surgery	164 (50%)	122 (48%)		
Active surveillance or watchful waiting	77 (23%)	67 (27%)		
Radiation therapy	72 (22%)	48 (19%)		
Androgen deprivation therapy =/‐ chemotherapy	10 (3%)	11 (4%)		
Other or missing	8 (2%)	4 (2%)		
Risk Score (National Comprehensive Cancer Network)			0.5	N/A
Missing	122 (37%)	84 (33%)		
Low risk	35 (11%)	28 (11%)		
Intermediate risk	108 (33%)	97 (38%)		
High risk	49 (15%)	27 (11%)		
Very high risk and metastatic	17 (5%)	16 (6%)		
EPIC‐26 Domain Summary Scores				N/A
Surgery			0.7	
Urinary incontinence	75.0	77.0		
Urinary irritative	91.5	90.7		
Bowel function	94.6	94.6		
Sexual function	28.4	29.7		
Radiation therapy			0.9	
Urinary incontinence	86.8	84.9		
Urinary irritative	84.6	82.5		
Bowel function	89.1	89.1		
Sexual function	25.3	28.2		
Active surveillance or watchful waiting			0.72	
Urinary incontinence	92.2	90.6		
Urinary irritative	92.6	87.7		
Bowel function	94.2	94.8		
Sexual function	49.7	52.0		
ADT =/‐chemotherapy			0.73	
Urinary incontinence	82.1	88.6		
Urinary irritative	86.9	82.5		
Bowel function	93.8	90.8		
Sexual function	10.1	15.6		
Other/missing			0.22	
Urinary incontinence	91.2	97.9		
Urinary irritative	90.6	81.3		
Bowel function	97.9	88.5		
Sexual function	37.0	27.5		

### 3.2 Factors Associated With Consent Status

Men who consented to accessing BroSupPORT were similar to those who chose not to consent. No statistically significant differences were found for remoteness, NCCN risk group score or EPIC‐26^12^ HRQoL domain scores. In addition, no overall relationship between consent rate and the SEIFA index was found (*p* = 0.36). In the multivariable analysis, after adjusting for men with missing NCCN scores (*n* = 222), being diagnosed in a public hospital was an independent predictor of consent status (OR: 0.62; 95% CI: 0.39–0.97). Remoteness, age, NCCN risk group, treatment type and EPIC‐26^12^ HRQOL domains were all excluded from the model as they were not associated with consent status. Although the SEIFA showed an overall association (*p* = 0.05) in the multivariable analyses, the relationship was not linear and each quintile's confidence interval passed through 1, meaning that there was insufficient evidence to conclude that the groups are significantly different. Sensitivity analysis including men with missing NCCN risk group scores resulted in SEIFA no longer being significantly associated with participating in BroSupPORT (Table S1).

### 3.3 Portal Activity

Of the 331 men consenting to the study, 253 (76%) accessed the comparator tool, and 114 (45%) continued to the supporting articles and resources. On average, men spent 3min and 20s on the portal per session, and 23% of men returned to the portal a second time. Seventy‐seven percent of men who accessed the portal scrolled to the bottom of each page. Men were most interested in learning more about sexual function (28%) and urinary issues (26%).

### 3.4 Portal Feedback

Of those who completed the exit survey in HotJar (*n* = 24), 17 (71%) found the comparator tool useful or very useful (Table 2). All respondents found the information to be easy or very easy to understand (20/20), and the articles were either useful or very useful (17/17). The Qualtrics survey, distributed 2 weeks after men consented to participate in BroSupPORT, was completed by 105/331 (32%) men. Few responding men (7/36) reported watching the videos, and of these, most (6/7) found them helpful or very helpful (Table 2). Most men found the portal easy to use, reassuring and informative. Although most men had no specific dislikes, 9% of men found the comparisons difficult to interpret. Men's suggestions for improving the portal included providing more specific local information, a search feature, explanations on how to interpret the comparisons and extending access beyond 30 days. Detailed responses to each question are outlined in Table 3.

**Table 2:** Survey feedback obtained upon portal exist (Hotjar).

Question	Count (percent)
How useful has the comparator tool been for you?	(*n* = 24)
Not at all useful	1 (4%)
Slightly useful	3 (13%)
Moderately useful	3 (13%)
Very useful	6 (25%)
Extremely useful	11 (46%)
How easy was it to understand the information provided?	(*n* = 20)
Extremely difficult	0
Slightly difficult	0
Moderately difficult	0
Easy	8 (40%)
Very easy	12 (60%)
How useful was the article you just read?	(*n* = 17)
Not at all useful	0
Slightly useful	0
Moderately useful	0
Very useful	9 (53%)
Extremely useful	8 (47%)
How informed do you feel after visiting this website?	(*n* = 6)
I feel a lot more informed	1 (17%)
I feel a little more informed	5 (83%)
I don't feel any more informed	0
How likely are you to take action based on the information you've read so far?	(*n* = 6)
Unlikely	0
Unsure	2 (33%)
Likely	3 (50%)
Very likely	1 (17%)

**Table 3:** Qualitative survey feedback obtained through the follow‐up survey administered 2 weeks after consent.

Question and response	Number of respondents
What did you like about the portal? Ease of use and informative content: The most appreciated aspect of the portal was its user‐friendly interface and the clarity of the information provided. About 45% of respondents found it easy to use and understand. Reassurance and informative value: 26% of users felt reassured by the portal, particularly in understanding how they compared to other men, while 22% found the portal informative. Potential for future use: 4% expressed an intention to return to the portal in the future. Pretreatment value: A small portion (1%) wished they had access to the portal before making treatment decisions. Perceived worth: Another 1% found the portal's report valuable for comparing their journey with others.	**76**
What did not you like about the portal? No specific dislikes: A majority (73%) did not have specific dislikes, indicating general satisfaction. Interpretation challenges: 9% struggled with interpreting the comparisons provided by the portal. Unclear purpose: 3% were unsure about the overall goal of the portal. Other issues: Smaller percentages reported issues such as poor design, limited access time, technical errors (e.g., PDFs cutting through graphs, error messages) and content that was heavily focused on late‐stage cancer patients. In addition, 3% mentioned feeling depressed after using the portal.	**67**
How would you improve the portal? Uncertainty in improvement: 73% were either unsure of how to improve the portal or were happy with it as it is. Enhancing information and functionality: Suggestions included providing more explicit information about the portal's aims and local data (5%), adding a search function (2%), fixing technical issues like error messages (2%) and offering better explanations of data and comparisons (3%). Extended access and reporting: 3% wanted earlier access to the portal in their care process, and another 3% suggested extending access beyond 30 days. There was also interest in the ability to generate formal reports (2%). Cultural relevance and alternative information: Respondents recommended including images of Australian men (2%) and more information on alternative medicines (2%). In addition, 2% suggested incorporating comorbidity comparisons.	**62**

## 4 Discussion

This is the first study, to our knowledge, to develop, pilot and evaluate a co‐designed online support portal for men with PCa and to assess the acceptability of integrating a PRO comparator tool within a portal on a whole‐of‐population scale. Qualitative research demonstrated that the BroSuPORT portal was generally well accepted by users and that men spent, on average, a long time on the portal with some returning to the website.

Our finding that BroSuPORT access was highly skewed towards men living in areas associated with greater socioeconomic status is concordant with literature examining the use of web portals more broadly. Studies suggest that socioeconomic status, including higher education and income, is associated with increased engagement with internet‐based technologies, particularly for cancer patients [20, 21, 22, 23]. This finding likely reflects the inclusion criteria for our study, which are being English‐speaking and having access to the internet at home. Further work is required to recruit more diverse men and make the portal available in other languages, to make portal access more equitable.

This study demonstrated that consent and engagement with the portal did not vary by men's location of residence, treatment type, cancer severity, age, or EPIC‐26^12^ results. Despite a greater number of men accessing the portal after being diagnosed in a private hospital, we found that men with PCa were more likely to consent to the portal if they were diagnosed in a public hospital. This may indicate that men diagnosed through private hospitals have greater access to support information and resources and thus may feel less inclined to access this information through an online portal than men diagnosed through a public hospital. There is evidence that there are differences in PCa service provision and treatment choice based on the type of health system men access [24]. Men diagnosed in the private health system are primarily supported by their urologist, whereas men diagnosed in the public system are often seen by multiple clinicians and rely on multidisciplinary support [25]. It may be that this bias indicates a need to improve supportive care services and resource provision to these men, especially after treatment for PCa.

An unexpected finding of the study was the small percentage of men who watched the video stories of Australian men with PCa, especially given the high level of demand for video stories expressed both by men participating in co‐design workshops during Phase 1 and the literature suggesting that patient narratives are perceived as a helpful resource [26, 27]. This reflects differences between expressed preference and revealed behavior. The positioning of the videos at the bottom of the page, ‘below the fold’, may have contributed to poor viewership. Furthermore, a lack of cultural diversity may have also contributed to poor viewership, with the three men being of a white Anglo‐Saxon background. This bias is the result of our recruitment approach, using members of the co‐design workshops who were predominantly of Anglo‐Saxon background. Further research is required to understand the impact of repositioning videos within the portal and widening the diversity of representation in videos.

A significant limitation of the study was the bias in access to the study. Access was limited to men who spoke English, had access to the internet and a computer at home and completed the EPIC‐26^12^ questionnaire via email, making it difficult to ascertain how other men may engage with an online portal. This undoubtedly compromises the generalisability of our findings for men with PCa in Victoria; however, 70% of all participants in the registry complete their PROM via email. Future research should examine the barriers and enablers to portal access for men to implement culturally appropriate and accessible solutions that enable their supportive care needs to be met. Another limitation of our study was that members of the co‐design team who played a major role in development and user acceptability testing may not be representative of the population of men for whom the portal was developed. This cohort contains men who are likely to be more informed about PCa and proficient with the use of technology in comparison with other men with PCa in Victoria. In the future, we will capture user characteristics and incorporate feedback into the portal, to better identify and address areas where the portal may fall short of meeting users’ needs and expectations. Finally, due to disruptions in data collection caused by COVID‐19 restrictions in Victoria, some data pertaining to risk group and treatment were not collected at the time the men completed their PROM. This meant that for some patients, the comparison tool could not be incorporated into their feedback, resulting in some men experiencing error messages on the portal when attempting to use this function. Future studies should aim to understand the wider impact of a portal on catalysing action by men to improve their QoL. This is an important initial step in achieving this ultimate goal.

## 5 Conclusion

Patient portals enabling men to compare their PROs with other similar men, as well as provide access to information and resources, show promise in addressing the complex supportive care needs of men with PCa. As the first of its kind in Australia, the portal offers an efficient mechanism for men to gain access to supportive care information and resources that they may not have otherwise been afforded, as well as the ability to discover how their PROs compare to similar men after treatment for PCa. Embedding recruitment to BroSupPORT in the population‐based PCOR‐Vic not only ensures that it reaches a wide geographic audience in Victoria but also offers the potential to be modified for use by other clinical quality registries capturing PROs. We look forward to future developments of the portal as well as the implementation of similar solutions that harness the power of electronic PROM capture.

## Author Contributions

**Benjamin Shemesh:** writing–original draft, writing–review and editing, project administration. **Jacinta L. Opie:** writing–original draft, funding acquisition, project administration, supervision. **Rodney L. Dunn:** conceptualisation, methodology, validation, data curation. **Garth Mclaughlin:** project administration. **Vivek Argawal:** project administration, visualisation, validation, methodology. **Amanda Pomery:** project administration. **Chris Mac Manus:** project administration, data curation, software. **Colin O'Brien:** conceptualisation, supervision. **Katrina Lewis:** conceptualisation, supervision. **Max Shub:** conceptualisation. **Paula Wilton:** funding acquisition, conceptualisation, project administration. **Prassannah Satasivam:** project administration. **Melanie Evans:** methodology, validation, data curation, software, project administration. **Jeremy Millar:** writing–original draft, writing–review and editing, conceptualisation, investigation, methodology, validation, visualisation, project administration, supervision, funding acquisition. **Sue M. Evans:** conceptualisation, investigation, funding acquisition, writing–original draft, methodology, validation, visualisation, writing–review and editing, project administration, supervision.

## Ethics Statement

This study was approved by the Alfred Health Ethics Committee (59472, Project 714/9) and Monash University HREC (24688).

## Consent

The authors have nothing to report.

## Conflicts of Interest

The authors declare no conflicts of interest.

## Patient or Public Contribution

The patient portal piloted in this study was developed using a co‐design approach including men with prostate cancer and health professionals involved in prostate cancer care. Men with prostate cancer formed part of the study's steering committee and consumer advisory groups. In addition, men with prostate cancer, their support persons and health professionals were involved in the development of the portal. Men with prostate cancer were also involved in the preparation of this manuscript.

## Supporting information

Supporting information.

## Data Availability

The datasets during and/or analysed during the current study are available from the corresponding author upon reasonable request.

## References

[1] S. M. Gilbert , R. L. Dunn , D. Wittmann , et al., “Quality of Life and Satisfaction Among Prostate Cancer Patients Followed in a Dedicated Survivorship Clinic,” Cancer 121, no. 9 (2015): 1484–1491, 10.1002/cncr.29215. 25538017 PMC10792765

[2] A. J. L. King , M. Evans , T. H. M. Moore , et al., “Prostate Cancer and Supportive Care: A Systematic Review and Qualitative Synthesis of Men's Experiences and Unmet Needs,” European Journal of Cancer Care 24, no. 5 (2015): 618–634, 10.1111/ecc.12286. 25630851 PMC5024073

[3] E. Watson , B. Shinkins , E. Frith , et al., “Symptoms, Unmet Needs, Psychological Well‐Being and Health Status in Survivors of Prostate Cancer: Implications for Redesigning Follow‐Up,” BJU International 117, no. 6B (2016): E10–E19, 10.1111/bju.13122. 25818406

[4] J. L. Gore , L. Kwan , S. P. Lee , R. E. Reiter , and M. S. Litwin , “Survivorship Beyond Convalescence: 48‐Month Quality‐of‐Life Outcomes After Treatment for Localized Prostate Cancer,” JNCI: Journal of the National Cancer Institute 101, no. 12 (2009): 888–892, 10.1093/jnci/djp114. 19509365

[5] M. Wallace and S. Storms , “The Needs of Men With Prostate Cancer: Results of a Focus Group Study,” Applied Nursing Research 20, no. 4 (November 2007): 181–187, 10.1016/j.apnr.2006.08.008. 17996804

[6] L. Bourke , S. A. Boorjian , A. Briganti , et al., “Survivorship and Improving Quality of Life in Men With Prostate Cancer,” European Urology 68, no. 3 (September 2015): 374–383, 10.1016/j.eururo.2015.04.023. 25941049

[7] M. S. Litwin , D. P. Lubeck , J. M. Henning , and P. R. Carroll , “Differences in Urologist and Patient Assessments of Health Related Quality of Life in Men With Prostate Cancer: Results of the CaPSURE Database,” Journal of Urology 159, no. 6 (June 1998): 1988–1992. 9598504 10.1016/S0022-5347(01)63222-1

[8] F. Efficace , M. Feuerstein , P. Fayers , et al., “Patient‐Reported Outcomes in Randomised Controlled Trials of Prostate Cancer: Methodological Quality and Impact on Clinical Decision Making,” European Urology 66, no. 3 (2014): 416–427, 10.1016/j.eururo.2013.10.017. 24210091 PMC4150854

[9] L. Warrington , K. Absolom , and G. Velikova , “Integrated Care Pathways for Cancer Survivors: A Role for Patient‐Reported Outcome Measures and Health Informatics,” Acta Oncologica 54, no. 5 (May 2015): 600–608, 10.3109/0284186x.2014.995778. 25751758

[10] J. M. Valderas , A. Kotzeva , M. Espallargues , et al., “The Impact of Measuring Patient‐Reported Outcomes in Clinical Practice: A Systematic Review of the Literature,” Quality of Life Research 17, no. 2 (2008): 179–193, 10.1007/s11136-007-9295-0. 18175207

[11] S. M. Evans , J. L. Millar , J. M. Wood , et al., “The Prostate Cancer Registry: Monitoring Patterns and Quality of Care for Men Diagnosed With Prostate Cancer,” BJU International 111, no. 4 Pt B (2013): E158–E166, 10.1111/j.1464-410X.2012.11530.x. 23116361

[12] J. T. Wei , R. L. Dunn , M. S. Litwin , H. M. Sandler , and M. G. Sanda , “Development and Validation of the Expanded Prostate Cancer Index Composite (EPIC) for Comprehensive Assessment of Health‐Related Quality of Life in Men With Prostate Cancer,” Urology 56, no. 6 (2000): 899–905, 10.1016/s0090-4295(00)00858-x. 11113727

[13] B. Shemesh , J. Opie , E. Tsiamis , et al., “Codesigning a Patient Support Portal With Health Professionals and Men With Prostate Cancer: An Action Research Study,” *Health Expectations* 25, no. 4: 1319–1331, 10.1111/hex.13444. PMC9327875 35411697

[14] A. L. Hartzler , J. P. Izard , B. L. Dalkin , S. P. Mikles , and J. L. Gore , “Design and Feasibility of Integrating Personalized PRO Dashboards Into Prostate Cancer Care,” Journal of the American Medical Informatics Association 23, no. 1 (January 2016): 38–47, 10.1093/jamia/ocv101. 26260247 PMC5009933

[15] A. Fagerlin , B. J. Zikmund‐Fisher , and P. A. Ubel , “If I'm Better Than Average, Then I'm Ok?”: Comparative Information Influences Beliefs About Risk and Benefits,” Patient Education and Counseling 69, no. 1–3 (2007): 140–144, 10.1016/j.pec.2007.08.008. 17942271 PMC2189559

[16] D. E. Avison , F. Lau , M. D. Myers , and P. A. Nielsen , “Action Research,” Communications of the ACM 42, no. 1 (1999): 94–97.

[17] L. H. Hotjar , Hotjar Ltd 2024 [Software]. St. Julian's, Malta, accessed November 16, 2021, https://www.hotjar.com/.

[18] National Comprehensive Cancer Network , NCCN Guidelines‐ Prostate Cancer, 2022, https://www.nccn.org/guidelines/guidelines-detail?category=1&id=1459.

[19] Australian Bureau of Statistics, *Census of Population and Housing: Socio‐Economic Indexes for Areas (SEIFA)*, Catalogue No. 2033.0.55.001 (Australia: Australian Bureau of Statistics, 2016), https://www.abs.gov.au.

[20] A. Girault , M. Ferrua , B. Lalloué , et al., “Internet‐Based Technologies to Improve Cancer Care Coordination: Current Use and Attitudes Among Cancer Patients,” European Journal of Cancer 51, no. 4 (March 2015): 551–557, 10.1016/j.ejca.2014.12.001. 25661828

[21] R. L. Kruse , R. J. Koopman , B. J. Wakefield , et al., “Internet Use by Primary Care Patients: Where is the Digital Divide?,” Family Medicine 44, no. 5 (May 2012): 342–347. 23027117

[22] E. S. Rodriguez , “Using Patient Portals to Increase Engagement in Patients With Cancer,” Seminars in Oncology Nursing 34, no. 2 (May 2018): 177–183, 10.1016/j.soncn.2018.03.009. 29625803

[23] W. S. Chou , B. Liu , S. Post , and B. Hesse , “Health‐Related Internet Use Among Cancer Survivors: Data From the Health Information National Trends Survey, 2003–2008,” Journal of Cancer Survivorship 5, no. 3 (September 2011): 263–270, 10.1007/s11764-011-0179-5. 21505861

[24] P. D. Baade , R. A. Gardiner , M. Ferguson , et al., “Factors Associated With Diagnostic and Treatment Intervals for Prostate Cancer in Queensland, Australia: A Large Cohort Study,” Cancer Causes & Control: CCC 23, no. 4 (April 2012): 625–634, 10.1007/s10552-012-9931-z. 22382868

[25] L. te Marvelde , R. L. Milne , C. J. Hornby , A. B. Chapman , G. G. Giles , and I. E. Haines , “Differences in Treatment Choices for Localised Prostate Cancer Diagnosed in Private and Public Health Services,” Medical Journal of Australia 213, no. 9 (2020): 411–417, 10.5694/mja2.50794. 32996611

[26] J. Engler , S. Adami , Y. Adam , et al., “Using Others’ Experiences. Cancer Patients’ Expectations and Navigation of a Website Providing Narratives on Prostate, Breast and Colorectal Cancer,” Patient Education and Counseling 99, no. 8 (August 2016): 1325–1332, 10.1016/j.pec.2016.03.015. 27067064

[27] B. J. Davison , M. Keyes , S. Elliott , J. Berkowitz , and S. L. Goldenberg , “Preferences for Sexual Information Resources in Patients Treated for Early‐Stage Prostate Cancer With Either Radical Prostatectomy or Brachytherapy,” BJU International 93, no. 7 (2004): 965–969, 10.1111/j.1464-410X.2003.04761.x. 15142144

