# Supplementary material for: Developing, Piloting and Evaluating a Patient Support Portal for Men With Prostate Cancer in Victoria: An Action Research Study
Source: Health Expect. 2025 Jan 19;28(1):e70149. doi: 10.1111/hex.70149 (PMC11743188; doi:10.1111/hex.70149)
Supplement: Supplementary file 1 — Supporting information. [file HEX-28-e70149-s001.docx]

## Supplementary table 1.

**Sensitivity Analysis**

| **Sensitivity Analysis**  **Variable** | **Level** | **Consent rate** | **Odds Ratio [95% CI]** | **P-Value** |
| --- | --- | --- | --- | --- |
| Diagnosing institute | Public | 120/186 (64.5%) | 1 (reference) | 0.018 |
|  | Private | 199/368 (54.1%) | 0.65 [0.45 - 0.93] |  |
| **Non-Significant variables excluded from the final model** | | | | |
| SEIFA  Disadvantage Quintile | 3 | 60/110 (54.5%) | 1 (reference) | 0.065 |
|  | 1 | 17/42 (40.5%) | 0.57 [0.27 - 1.17] |  |
|  | 2 | 51/85 (60.0%) | 1.26 [0.71 - 2.25] |  |
|  | 4 | 70/106 (66.0%) | 1.63 [0.94 - 2.84] |  |
|  | 5 | 121/211 (57.3%) | 1.13 [0.71 - 1.80] |  |
| Age group | <=59 | 48/83 (57.8%) | 1 (reference) | 0.371 |
|  | >=75 | 67/109 (61.5%) | 1.19 [0.66 - 2.14] |  |
|  | 60-64 | 46/93 (49.5%) | 0.69 [0.38 - 1.26] |  |
|  | 65-69 | 84/144 (58.3%) | 1.04 [0.60 - 1.81] |  |
|  | 70-74 | 74/125 (59.2%) | 1.12 [0.63 - 1.97] |  |
| Remoteness | 1 | 209/361 (57.9%) | 1 (reference) | 0.364 |
|  | 2 | 90/151 (59.6%) | 1.06 [0.72 - 1.57] |  |
|  | 3 | 20/42 (47.6%) | 0.65 [0.34 - 1.24] |  |
| Urinary Irritative | Per unit increase | 554 | 1.00 [0.99 - 1.02] | 0.550 |
| Urinary Incontinence | Per unit increase | 554 | 1.00 [0.99 - 1.01] | 0.649 |
| Treatment type | Surgery | 158/273 (57.9%) | 1 (reference) | 0.711 |
|  | ADT +/- Chemotherapy | 9/19 (47.4%) | 0.66 [0.26 - 1.69] |  |
|  | Other or Missing | 8/12 (66.7%) | 1.53 [0.45 - 5.21] |  |
|  | Radiation Therapy | 70/115 (60.9%) | 1.14 [0.73 - 1.78] |  |
|  | WWAS | 74/135 (54.8%) | 0.90 [0.59 - 1.36] |  |
| NCCN Risk group score | 1 | 34/59 (57.6%) | 1 (reference) | 0.562 |
|  | 2 | 107/197 (54.3%) | 0.86 [0.48 - 1.56] |  |
|  | 3 | 48/74 (64.9%) | 1.33 [0.66 - 2.69] |  |
|  | 4 | 16/31 (51.6%) | 0.79 [0.33 - 1.91] |  |
|  | Missing | 114/193 (59.1%) | 1.06 [0.59 - 1.92] |  |
| Bowel function | Per unit increase | 554 | 1.00 [0.99 - 1.02] | 0.789 |
|  | Per unit increase | 554 | 1.00 [0.99 - 1.00] | 0.353 |
